# A Context-Aware Indoor Air Quality System for Sudden Infant Death Syndrome Prevention

**DOI:** 10.3390/s18030757

**Published:** 2018-03-02

**Authors:** Daniel H. De La Iglesia, Juan F. De Paz, Gabriel Villarrubia González, Alberto L. Barriuso, Javier Bajo

**Affiliations:** 1 BISITE Digital Innovation Hub., University of Salamanca, Edificio Multiusos I+D+i, C/Espejo SN, 37007 Salamanca, Spain; fcofds@usal.es (J.F.D.P.) gvg@usal.es (G.V.G.); albarriuso@usal.es (A.L.B.); 2 Artificial Intelligence Department, Polytechnic University of Madrid, Campus Montegancedo s/n, Boadilla del Monte, 28660 Madrid, Spain; jbajo@fi.upm.es

**Keywords:** context-aware, SIDS, non-intrusive, e-health, pediatric

## Abstract

Context-aware monitoring systems designed for e-Health solutions and ambient assisted living (AAL) play an important role in today’s personalized health-care services. The majority of these systems are intended for the monitoring of patients’ vital signs by means of bio-sensors. At present, there are very few systems that monitor environmental conditions and air quality in the homes of users. A home’s environmental conditions can have a significant influence on the state of the health of its residents. Monitoring the environment is the key to preventing possible diseases caused by conditions that do not favor health. This paper presents a context-aware system that monitors air quality to prevent a specific health problem at home. The aim of this system is to reduce the incidence of the Sudden Infant Death Syndrome, which is triggered mainly by environmental factors. In the conducted case study, the system monitored the state of the neonate and the quality of air while it was asleep. The designed proposal is characterized by its low cost and non-intrusive nature. The results are promising.

## 1. Introduction

Due to the recent advances in sensor systems, the Internet-of-Things, and medical devices, it is possible to provide personalized and continuous health care at home [1]. People with chronic diseases or elderly people are the ones that most benefit from these home care systems [2]. Thanks to these developments, patients can be more independent and reduce their visits to the doctor. This contributes greatly to the quality of their life [3]. The evolution of monitoring systems entails sensors collecting greater amounts of data; this means that we have to be prepared to process more medical data [4].

It is therefore essential to find solutions that will allow for the management of large amounts of data in a fast, efficient, and accurate way [5]. To achieve efficient management, it is essential to apply a context layer to the collected data. Context-aware systems play a notable role in the processing and analyzing of data [6]. The correct contextualization of the collected information is essential for understanding it and generating knowledge that can be used in decision making. To contextualize data, it is necessary to have additional information on the context that will make the collected data coherent and reliable [7]. In many cases, additional context information comes from heterogeneous data sources, and it is necessary that they are reliable and accurate context providers. Poorly contextualized datasets can lead intelligent applications and systems to make the wrong decisions. This is a very serious issue for medical systems, in which a wrong decision caused by a contextualization error can put the health of the patient at risk [8]. Hence, when designing homecare systems, it is important to pay close attention to the performance of the systems that add and manage contexts in context-aware systems.

Another important issue in today’s homecare systems is environmental factors such as air quality. It has been demonstrated that acting over these factors is a determinant in the prevention of different diseases [9]. The problem is that much of current sensor systems employ only biomedical measurement devices that users must carry with them or with which they must interact in some way (such as a pulse sensor, body temperature meter, or glucometer). Just a few projects implement systems that monitor environmental factors in the home.

The lack of research in this area creates a necessity for new proposals in the field of air quality monitoring systems in homecare. These systems must measure air quality in domestic environments with the aim of improving user’s health. Current systems are compatible with traditional health care systems, based on biomedical sensors. The combination of both systems ensures the accuracy of information; this allows the prevention or treatment of certain diseases more effectively [10]. In some cases, the use of biomedical sensors may be highly intrusive for the patient, and experts only recommend the use of air quality monitoring systems. One of these cases is the SIDS (Sudden Infant Dead Syndrome) [11], studied in this work. We propose a system for the monitoring of air quality factors that trigger SIDS. This health problem (also known as “crib death” or “cot death”) is the death of a child under the age of one, which occurs abruptly and unexpectedly and remains unexplained after a post-mortem study. However, placing medical sensors on the body of the newborn increases the chances of it suffering this syndrome. Death usually occurs during sleep [12]. Associated with SIDS is the ALTE (An Apparently Life-Threatening Event) that occurs when the infant has a prolonged sleep apnea crisis; it exhibits altered muscle tone and skin color. The infant does not respond to small stimuli until cardiopulmonary resuscitation is performed [13].

In developed countries, SIDS is one of the most frequent causes of mortality in infants aged between 28 days and one-year-old. It is the third leading cause of death in infants in the US (8%) and the most frequent in the post-neonatal period (40–50%). In 90% of cases it occurs during the first 6 months of life. Maximum incidence occurs between the 2nd and 4th month; it is infrequent in the first month of life, sporadic after six months, and exceptional after one year. The incidence of SIDS has decreased thanks to preventive actions; in most European countries it occurs 1.5 and 2 per 1000 births. In countries like Spain, mortality rates are 0.34 per 1000 births [12]. The incidence is higher in males and in cold and wet months. This is due to excessive bedding along with excessive temperature and humidity in the room. The risk is greater if the infant weighs less than 1500 g [13].

On the other hand, the incidence of ALTE in the United States is estimated to occur in 0.5–6% of newborns. Some studies suggest that between 5–10% of SIDS victims had previously had an ALTE episode [14]. At present, SIDS has not been found to present any pathognomonic aspect (a symptom that characterizes and defines a certain disease). It is a multifactorial process that does not obey a specific and unique cause [12]. However, the most accepted hypothesis associates this syndrome with a cardiorespiratory control disorder in the brainstem, causing alterations in arterial tension and in the sleep wake cycle. Alterations in respiratory regulation during sleep lead to a ventilation deficit. Babies that are at a higher risk of suffering SIDS have a lower capacity for ventilator response to hypercapnia or hypoxia, which trigger progressive respiratory depression. All this, together with external factors (e.g., viral infections, exposure to tobacco smoke, and ambient stressors) can lead to death [15].

In recent years, other risk factors like air quality, temperature, or humidity have been identified; they are related to the environment in which the child lives. It is possible to act positively with regard to these factors and, therefore, decrease the frequency of SIDS, as demonstrated in [16]. The monitoring of these factors is essential for preventing this pathogen from causing the baby’s death during sleep [17,18].

This paper presents a system that integrates different air quality sensors, together with real-time image processing techniques, for the monitoring of the main air quality factors that cause SIDS (temperature, humidity, and gases concentration). By analyzing the air quality in the room where the infant sleeps, it is possible to detect early risks that may affect them [19]. The system includes a module that monitors the baby’s heart rate through the analysis of the images obtained by a webcam. Thanks to real-time heart rate monitoring, it is possible to monitor the health of the newborn and prevent possible episodes of SIDS. A relevant aspect of the work is that it proposes a non-intrusive system, since no sensor is in direct contact with the baby’s body. All the data generated by the sensor system is managed and contextualized by a context-aware framework. This framework is capable of generating knowledge that allows the detection of anomalies in the data. This article aims to establish a safer sleeping environment for newborns, reducing the risk factors involved in SIDS.

The article is organized as follows: Section 2 overviews the state of the art in this area. Section 3 describes the designed context-aware system by the authors in this paper. Section 4 describes the proposed non-intrusive monitoring system and outlines the case study carried out in this work in a real environment. Finally, Section 5 presents the conclusions drawn from the conducted research and experiments.

## 2. Previous Works

As described in the previous section, the objective of this work is the design of an air quality monitoring system that is capable of detecting risks that lead to sudden death in infants. Before designing the system, it was necessary to review the current state of the art on the topic of the work. Context-aware systems are key tools for managing and generating knowledge from the data collected by sensor systems. In this section, we look at the main developments in monitoring and sensor systems for telemedicine and homecare systems. We also review the main context-aware systems proposed in the bio-sanitary and environmental monitoring fields. Finally, studies that address Sudden Infant Death Syndrome detection are considered.

### 2.1. Health Care Monitoring Systems

Health systems and telemedicine are important areas for the application of the Internet-of-Things (IoT) [20]. The capabilities provided by IoT have been used in medical applications such as remote health control, fitness programs, chronic disease treatment, or elderly care [21]. In recent years, researchers have worked with different IoT technologies, focusing on the field of health and the resolution of a variety of practical challenges. A very popular line of research among different research groups focused on Environmental Intelligence is Ambient Assisted Living (AAL). AAL systems are destined to make elderly people independent in their own home and in a comfortable and safe way. In the study [22], authors propose a modular architecture for automation, security, control, and communication in an AAL system. This framework provides different services to the elderly through the implementation of a radiofrequency-based communication system together with algorithms for the detection of medical problems managed by a human expert. The authors of [23] propose an open, flexible, and secure IoT cloud platform. This platform manages numerous limitations associated with interoperability, security, quality of service (QoS), and storage of medical data. The authors of [24] see this definition in AAL-based systems as fundamental. Other studies such as [25] stressed that a standard technological framework should be developed for AAL systems.

Mobile health services (m-health) use mobile devices, medical sensors, and communication technologies to provide mobile healthcare services as described in [26]. The use of m-IoT-based services has been examined for non-invasive sensor environments for the measurement of glucose levels as described in the paper [27]. The authors of [28] propose a system that combines the use of physical sensing devices (body temperature, blood pressure, and glucose) with non-invasive sensing devices (movement in the home, electricity consumption, and bed or sofa occupancy).

Different medical problems have been studied by researchers in recent years. The monitoring of the level of urine excreted by a patient admitted to a medical center is one example. The authors of [29] propose the use of a low cost wireless sensing device capable of measuring the level of excreted urine in real time, which makes it possible to identify possible medical problems quickly. Another example of a studied medical problem is systems that measure the patients’ electrocardiogram. Various studies [30,31,32,33] analyzed different ECG (electrocardiography) monitoring techniques in IoT environments. There are also numerous studies that monitor patients’ body temperature remotely [34,35].

The non-intrusive system proposed in this work will be able to integrate easily with health care systems to provide information on air quality. In the current literature, there are no systems of this type within health care systems and that is why this work seeks to provide new work in this area. 

### 2.2. Context-Aware and E-Health

The joint use of context-aware systems and e-health systems [1] for the contextualization of medical data from sensors is a field that is widely studied by researchers [36,37]. In [6], the authors defined the term Smart Health as a combination between e-Health and context-aware systems in smart cities. The authors made an overview of the main elements involved in Smart Health and described the different challenges and opportunities offered by this technology. The authors of [38] proposed a remote health monitoring system, based on context-aware and wireless devices based on Bluetooth. Along the same lines, the authors of [39] designed an e-Health system for the home environment. The proposal featured a combination of medical sensors and social sensors (temperature, opening of a door, and presence). With the use of a context-aware framework, medical and social sensors’ data are fused in order to generate knowledge that is capable of detecting anomalous symptoms in user behavior. In [40], authors develop a context-aware system for the monitoring of a specific medical problem, in this case the continuous monitoring of an edema. There are works that try to identify and group the different context-aware architectures and the challenges they face in order to formulate a common frame of reference [41].

In this topic, it is also not possible to find important references in the current literature that combine the use of non-intrusive systems for monitoring the risks of SIDS with a context-aware framework that provides context and precision with regard to the captured data.

### 2.3. Systems for the Prevention of SIDS Risks

After analyzing the current state of the art, we identified different works that propose different approaches for the prevention of SIDS. We first researched invasive monitoring systems, which use wearable devices that are placed on the newborns body. In these studies, [42,43,44,45], the authors obtain data through different medical sensors. In [42], the authors present a band that the newborn wears on its chest; it is equipped with a temperature sensor, a pulse and breath sensor, and a motion sensor that is capable of determining the position in which he is sleeping. These sensors provide all the data needed to determine the child’s state of health. The authors of [43] made use of commercial wearable medical sensors to collect the biomedical data of the newborn, which is sent to a remote server.

A widely studied field in the prevention of SIDS is the detection and monitoring of infant breathing while they are asleep, with the aim of detecting apneas that lead to premature death. The studies that focus on this problem adapt two particular techniques. On the one hand, there are works that propose non-invasive monitoring [46,47,48,49,50] and use different methodologies for this purpose. The authors of [46] analyzed the images taken by a camera installed on the baby’s crib in order to detect breathing movements. The authors of [47] proposed the analysis of acoustic signals produced by the neonate; they were registered by a microphone installed in its cradle. In [47], the authors presented the NanoPulse Baby SleepGuard system. This device is able to monitor breathing and heart rate during sleep. The device is equipped with UWB (ultra-wide-band) radar technology that records variations in the movement of the baby’s body. Other studies, however, propose invasive systems in which the sensors are in contact with the body. The authors of [51] analyzed the effectiveness of an inductive sensor versus a capacitive sensor in monitoring sleep apnea.

Another field of work in the prevention and control of SIDS is the monitoring of the environment in which the newborn rests. In this line, the authors of [52] propose the use of a device that is capable of measuring the level of CO_2_ (Carbon Dioxide) exhaled by the newborn in its cradle. Thanks to the data provided by this sensor, the authors can detect possible breathing anomalies that can lead to an apnea episode. The authors of [53] combined the use of an infrared temperature sensor together with a breathing detection system through the analysis of the acoustic signal collected by a microphone.

In the current literature, it is possible to find works that focus on the design of intelligent systems; they aim to provide accuracy in detecting possible alerts or incidents inferred from the data collected by sensor systems such as those described in this section. In [54], the authors presented a two-level approach based on neural networks that is capable of differentiating false alarms from real incidents.

Once the current literature on SIDS detection and monitoring systems has been analyzed, it is possible to identify a lack in works related to prevention based on monitoring through environmental sensors. The studied articles focus on the use of medical sensors for monitoring the state of health of newborns, but not for prevention based on environmental factors. Therefore, the aim is to provide a novel solution through the design of an environmental monitoring system and a Context-Awareness framework. It is a non-invasive system, unlike the vast majority of works found in the literature.

## 3. Proposed Framework

This section describes the designed context-aware framework. Its aim is to monitor domestic air quality conditions for the prevention of a specific health problem. Concretely, the proposed system monitors key risk factors that contribute to neonates suffering from Sudden Infant Death Syndrome (SIDS). The context-aware framework used in this work is based on the BDCaM (Big Data Context-Aware Monitoring) framework, for the remote monitoring of large amounts of medical data [55].

### 3.1. Background

As stated above, the BDCaM framework is specifically designed for the analysis of large medical data in cloud computing environments. BDCaM evolved from CoCaMAAL (Cloud-oriented Context-aware Middleware in Ambient Assisted Living) middleware [56], also proposed by the same authors. The aim of this CoCaMAAL is to process and administer large amounts of contexts obtained from different AAL systems. In the CoCaMAAL proposal, the authors describe the process of identifying context-aware services using high-level generalized medical rules. However, this model did not take full advantage of the potential of large amounts of patient data. This is because it did not generate personalized knowledge for patients. For this reason, the authors designed BDCaM as an extended version of the CoCaMAAL model. This new model includes learning and knowledge generation functionalities to find specific anomalies for each patient.

In [55], the authors implemented BDCaM in a medical case study in which knowledge was generated on changes in the users’ blood pressure and heart rate in different situations. In the case study, the authors also intended to find personalized rules for each patient and classify unknown situations on the basis of the generated learning models. 

In this work, the BDCaM framework is implemented in a non-intrusive monitoring environment for the measurement of air quality conditions that influence the health of newborns. Specifically, it is a platform that aids decision-making on the basis of the data collected by the sensorization system deployed in the newborn’s habitat. Air quality data are obtained in real time together with the infant’s heart rate, which is measured by a series of computer vision techniques through image analysis. Data from external heterogeneous sources are added to the collected data in order to correctly contextualize the data. Additional data include the geolocation of the neonate’s home, the weather in the area, and data from public open sources such as air quality and pollution levels in the environment.

An analysis of the current state of the art was carried out in this work when selecting a context-aware framework that would meet the needs of this proposal. One of the main references used in this analysis was the paper [41] in which authors described an exhaustive survey of the current state of the art in context-aware middleware architectures. Table 1 compares the principal candidates. As described before, in this work we opted for BDCaM. Its notable qualities include an innovative learning process in which connections between context attributes and threshold values are established. BDCaM is versatile and can be applied to different domains; it is interoperable and has a high fault tolerance.

BDCaM provides the tools needed to efficiently manage generated data. Moreover, it generates knowledge from contexts and past data stored in the system. All the aspects of the framework and its components are described in more detail in the next section.

### 3.2. Framework Description

**Figure 1:**
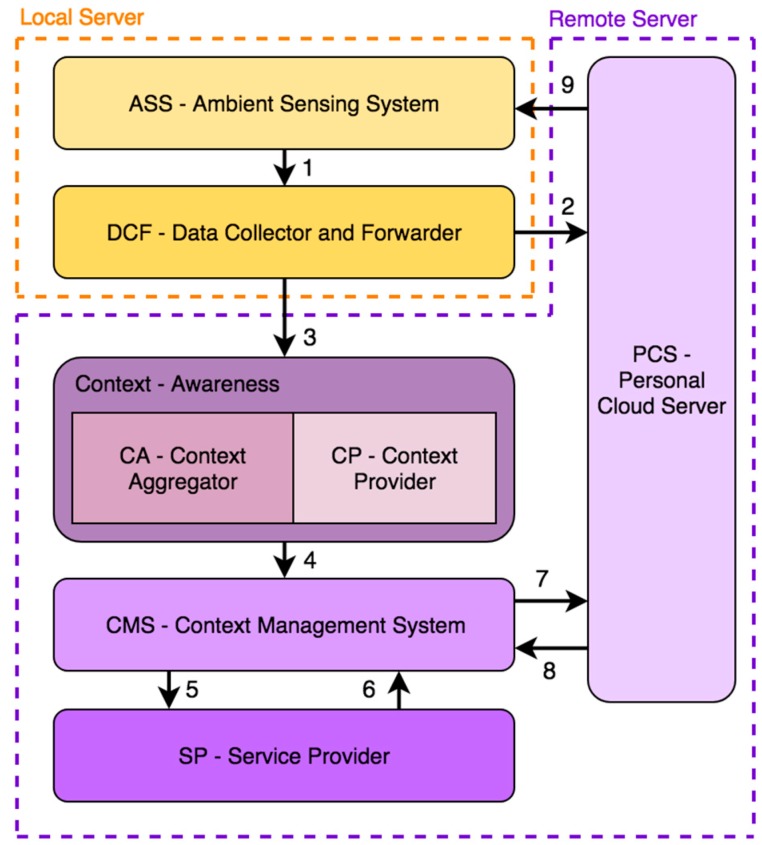
General diagram of the BDCaM architecture showing the main elements of the system, as well as the data flows between them: (**1**) raw data; (**2**) raw data for storage; (**3**) raw data to process; (**4**) context information and context states; (**5**) new general rule; (**6**) general rule; (**7**) new personalized rule or learned classifier; (**8**) personalized rules; and (**9**) warning, alert, or emergency.

The BDCaM framework, which will be the basis for system designed in this paper, is composed of different distributed elements, as illustrated in Figure 1. Furthermore, it is possible to observe the different data flows that occur between each one of its components. The architecture can be divided into four remote cloud components and two local components. The following subsections will describe these modules in detail.

#### 3.2.1. Ambient Sensing System

In the original architecture of the BDCaM model, this component is called AAL or Ambient Assisted Living. AAL systems are specially designed to prevent, cure, and improve the well-being and health of older people in domestic and residential settings [63]. These systems combine the use of air quality and medical sensors with other care tools such as medical treatment management systems or reminders to take medication [64,65]. In this way, older people can be more independent in dealing with their health. Our proposal modifies the BDCaM framework slightly, because the AAL system is replaced with a non-intrusive monitoring system. The Ambient Sensing (ASS) system is capable of generating a large amount of data. These data are generated by the monitoring of air quality through a WSN (Wireless Sensor Network) system and remote monitoring of the newborn through a non-intrusive heart rate calculation system.

To design this component, we have opted to implement a multi-agent system based on virtual organizations of agents that are capable of managing all the data collected by the deployed sensors. This technology is capable of dynamically creating agent organizations and programming tasks or behaviors, as well as establishing logical structures through relationships and roles [66]. As it is an open and self-organized architecture, it is possible to include different types of sensors that implement different types of communication (Bluetooth, Wi-Fi, RFID). Thus, from heterogeneous data it is possible to generate a set of low level data that is ready to be transmitted and processed.

**Figure 2:**
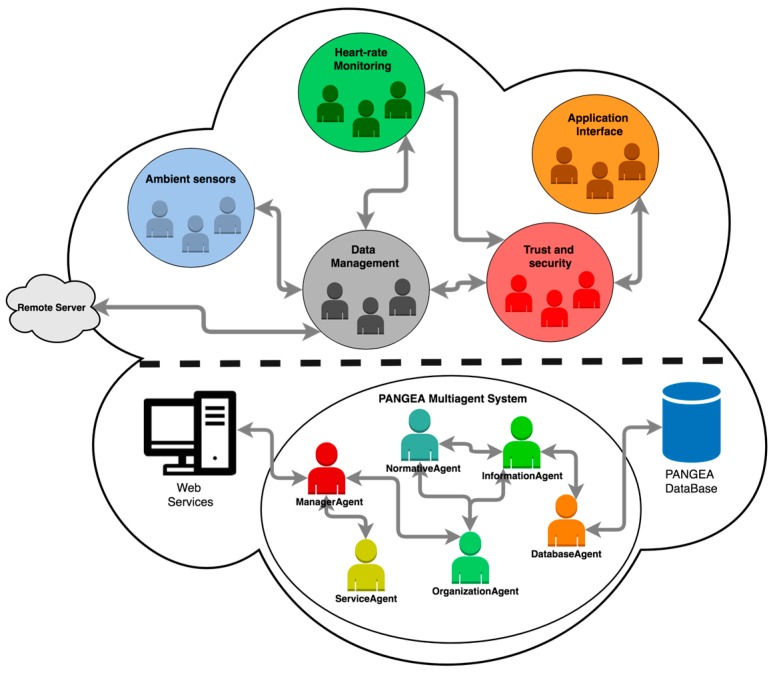
The virtual agent organization flow chart based on the PANGEA platform.

As it can be seen in Figure 2, the architecture is divided into two parts. The upper part contains the set of virtual organizations designed specifically for this work. The lower part of the architecture contains the multi-agent platform PANGEA (Platform for Automatic co-construction of orGanizations of intElligent Agents) [67]. From the range of principal platforms in current literature, such as JASON [68], JADE [69], or THOMAS [70], we chose PANGEA, because it is a simple, open, and dynamic platform. In addition, thanks to its low computational cost, it is possible to embed software agents in simple hardware devices such as sensors. PANGEA has been used in sensor systems deployed in various areas, such as sanitary [29], assistance [71], or agronomist [72].

The different virtual organizations specifically designed for this system are described below:

- *Ambient sensors organization*: As described above, in PANGEA it is possible to deploy software agents in simple hardware devices. This organization is composed of a set of agents embedded in the different sensors. There are two types of agents within this organization; one type is in charge of transforming the data obtained by the sensors in the physical layer. The other type includes coordinating agents in charge of transmitting this information to the rest of the organization.
- *Heart-rate monitoring organization*: This organization is responsible for managing the information captured by the HR (Heart-Rate) monitoring web camera and for taking and extracting images. It also processes the images to extract of heart-rate data. These data are sent to the trust and security organization in charge of ensuring the security of the data and the images obtained by the camera as they are highly sensitive data.
- *Data management organization*: It is the organization in charge of the management of all the data collected in the system. Both the ambient sensors organization and the heart-rate monitoring organization transmit their data to this coordinating organization. At this point, after obtaining the different data, the organization classifies and groups the data and prepares them for later transmission to the remote server in charge of processing them.
- *Trust and security organization*: This organization is in charge of the security and integrity of the different data captured. It is in charge of verifying the external access of the data to prevent unauthorized users or agents from accessing the data and images that have been generated. In addition, a verification of the integrity of the data is made to avoid the transmission of erroneous, corrupt, or null data.
- *Application Interface organization*: In this organization, the data that is visualized in the application layer of the system is managed. The organization is responsible for providing different data to different applications such as the mobile application or the web platform, adapting the raw data to the required format in each interface. It is coordinated with the trust and security organization to avoid, for example, providing live images of the HR monitoring camera to the applications that require them from outside the home local network. It is also in charge of displaying alert and emergency notifications that are issued from the central server.

#### 3.2.2. Data Collector and Forwarder

The traditional models of context-aware systems perform raw data processing (raw), produced by the sensor network, within the local network itself. This task is usually performed by mobile devices or small servers with low computing capacity, which process raw data to be later forwarded as high-level data to the corresponding remote server. In the case of the BDCaM model, the analysis of the large volumes of data generated by the sensor network is discarded locally, due to the limitations and lack of efficiency of these servers (such as a mobile device). Likewise, by keeping all the raw data of low level, it is possible to preserve all the data to perform on them different analyzes in later iterations without losing information in the process.

Therefore, in this module of the BDCaM architecture, a low-level data collected is carried out for its subsequent sending to the personal cloud server, where it will be stored, or to the context aggregation module for further analysis. In the case of study, this task is performed in the local server based on the Rasberry Pi board, which has Wi-Fi and Bluetooth connection and is capable of integrating other communication mechanisms such as ZigBee or RFID through external modules.

#### 3.2.3. Personal Cloud Server

It is a virtual server deployed on a dedicated cloud server (such as Amazon S3 or Microsoft HealthVault) that contains all the user information. Each of the different Ambient Sensinsing systems has a personal server. All low level raw data is stored here through the data collector module and *forwarder module*. In addition to these data, personal information of the user is stored, such as age, sex, pathologies, age of the mother, or if it is premature. All these data will be important for the generation of later knowledge. The servers implement restrictive security mechanisms to avoid external exposure of the data.

After the first analysis, the custom rules and patterns are stored in this server to be accessed quickly when the system requires it.

#### 3.2.4. Context-Awareness

The focus element of the BDCaM architecture is the context-awareness model. In order to guarantee the correct contextualization of the collected data, the architecture integrates the context aggregator module and the context provider module:

- *Context Aggregator (CA)*: This module is in charge of combining all the simple contexts in a single context state through the use of a context model. This element is key when it comes to relating all the context data that by themselves cannot provide any data or be interpreted in a wrong way. For example, if taken as a context, a significant increase in the temperature inside the home may seem to be a problem. But if this data is contextualized with an increase in the atmospheric temperature of the area where the house is located and a hot season of the year, the situation may become normal. Therefore, the past and present contextual information must be added when making a correct classification of the situation. After the completion of this contextual aggregation, the generated information is sent to the *Context Manager System module* for each specific user.
- *Context Provider (CP)*: It is a distributed cloud service that is the main source when generating contexts. The *Context Aggregator Module* performs a transfer of low-level raw data generated in the different Environments Sensing Systems to the Context Providers modules. These modules are responsible for applying well-known techniques on these data to generate an elementary context from low-level data. For example, by applying pattern recognition techniques, it is possible to determine the occupation or not of a room through the data collected by an acoustic sensor and an LDR (Light Dependent Resistor) sensor or to determine whether a newborn is asleep or awake by analyzing the HR monitor data. To obtain additional data to be used in the generation of basic contexts, the module makes use of different APIs and heterogeneous data sources available in the network. Some of the data from external sources that are used in the case study are:
  ○ *Geolocation services*: One of the user’s personal parameters that is stored in the system is the location of the home where it is displayed. In particular, GPS coordinates are stored. It is necessary to consult a geocoding service to obtain the necessary geographic data to consult the rest of the data, such as weather.
  ○ *Weather*: In an environmental monitoring system, the weather of the area is an important factor that can determine potentially dangerous situations of normal situations. The same value measured by an indoor temperature sensor can mean different things depending on the climatic conditions of the area where this value has been measured.
  ○ *Air quality*: This is another one of the important data in the environmental monitoring systems studied. Many small and large-scale governments around the world are now providing this type of open data to their citizens. The air quality in the area where the home of a newborn is located is a critical factor in the contextualization of the data of the different environmental sensors. If the home is located in an urban area with a high concentration of traffic in the environment, the values will be very different from another located in the countryside, far from the city.
  ○ *Atmospheric data history*: Not only the current values are important; also, the historical values of the environmental conditions of an area are significant when evaluating the data measured by the different sensors.

#### 3.2.5. Context Management System

It is a key component when extracting knowledge from all the data and contexts available in the system. The Context Management System (CMS) is composed of a series of distributed cloud servers that are responsible for managing the big data generated on the platform. In particular, at this point the history of the different contexts of all users is stored. On all these stored data, different machine learning techniques are executed to infer generic and customized rules for different events. When a new custom rule is discovered, it is sent and stored in that user’s personal cloud server. If a new generic rule is discovered, it is sent to the Cloud Service Provider (SP) component, which is explained below. In this way, the CMS is responsible for keeping the different components updated, with the latest knowledge rules generated.

Sometimes, the rules that have been generated previously are necessary when generating new and better high-level knowledge. To do this, the component accesses the general rules stored in the *Service Provider Component* and the custom rules that are stored in the *Personal Cloud Server*. In this way, the system is in a continuous process of review and improvement of the rules and knowledge previously generated.

After generating the corresponding rules, a second stage of training is carried out that makes use of different data mining algorithms in order to obtain the best possible classifier for that rule. Once the optimal precision is reached, this model will be executed on the new data that is coming from the sensorization system. In the case of detecting a possible situation of danger or anomaly, an alert is issued to the Ambient Sensing System (ASS) in order to notify the user of this incident.

#### 3.2.6. Service Provider

In the BDCaM model, the services providers are distributed cloud servers that store the generic medical rules for the identification of anomalous situations or emergencies. These rules are continually monitored and updated by human experts who may be experts in medicine, in air quality, or in pediatrics. Therefore, in this module of the system, all the knowledge generated in the Context Management System is supervised and updated through human knowledge.

## 4. Proposed Non-Intrusive Monitoring System

This section describes the proposed non-intrusive system for the detection of possible air quality risks that trigger sudden infant death syndrome. In the first place, the identified risk factors are described. It was necessary to consider these factors when designing the system. Second, the different sensors used in the case study are described. Moreover, we look at gas levels that are considered harmful by human experts. The heart rate calculation system is also described below; it obtains the infants heart rate by analyzing the images captured by the webcam. Finally, we overview the entire system and the results obtained in the case study.

### 4.1. Analysis of Risk Factors in SIDS

This section analyses the different risk factors involved in SIDS. First, all the elements related to the neonate are analyzed. All those factors that do not have to do with the atmospheric conditions have been discarded, since that is not the objective in this study. Table 2 shows the direct risk factors [73].

Out of these factors, those that do not require environmental monitoring such as: age, sex, race, etc., should be discarded. These data will be relevant when contextualizing the data measured by the sensor network. The monitoring of the position of the newborn while asleep has been ruled out in this work. The use of any sensor that registers the position requires the placement of a device in the baby’s body that would add a new external risk factor. The only non-intrusive method of monitoring this factor is through the analysis of the camera images, which will be studied in a future line of work.

The air quality factors that can be monitored in real time are exposure to tobacco smoke, the increase in the amount of CO_2_ in the environment as a result of sharing bed with parents or siblings, and the thermal stress produced by the heating systems together with an increase in humidity. In addition, a monitoring of the concentration of accumulated gases in the room where the newborn rests is carried out.

### 4.2. Sensorization System

To monitor the identified risk factors, it is necessary to implement specialized hardware that will support the different sensors. This device will act as the central monitoring node in the proposed sensor network. In the current market there are different types of devices that have this capacity. The Arduino platform is the most extended, free code, and reduced cost. Therefore, a board based on Arduino has been the device used in the design of the system.

**Figure 3:**
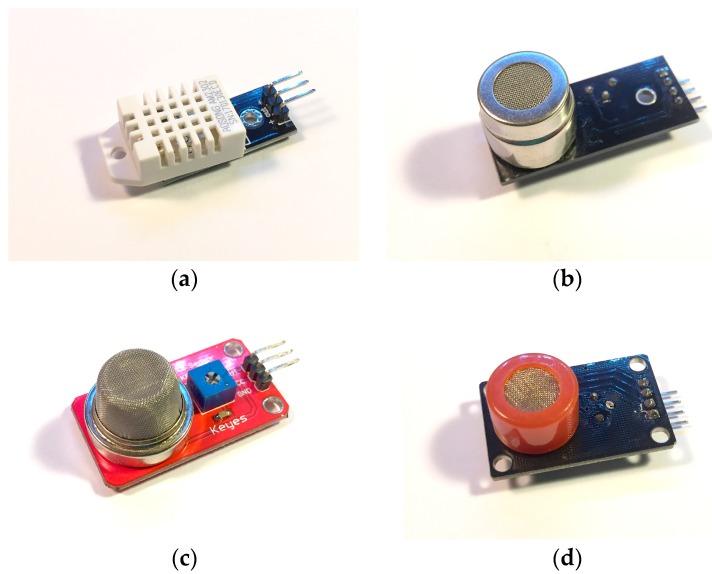
Low cost air quality sensors that make up the designed sensorization device: (**a**) the DHT22 sensor measures ambient temperature in addition to relative humidity of the air; (**b**) the purpose of the MG-811 sensor is to measure the concentration of carbon dioxide (CO_2_) in the environment; (**c**) the MQ-2 sensor measures smoke in the environment and is also sensitive to liquefied petroleum gas, propane, methane, alcohol and hydrogen; (**d**) the MQ-7 sensor is responsible for measuring the concentration of carbon monoxide (CO).

Next, Figure 3 describes the air quality sensors that will be part of the architecture. These sensors are low cost and have a high accuracy when measuring both temperature and humidity and the concentration of harmful gases. The sensors have been previously calibrated by the manufacturer and have an accuracy of ±1%.

**Figure 4:**
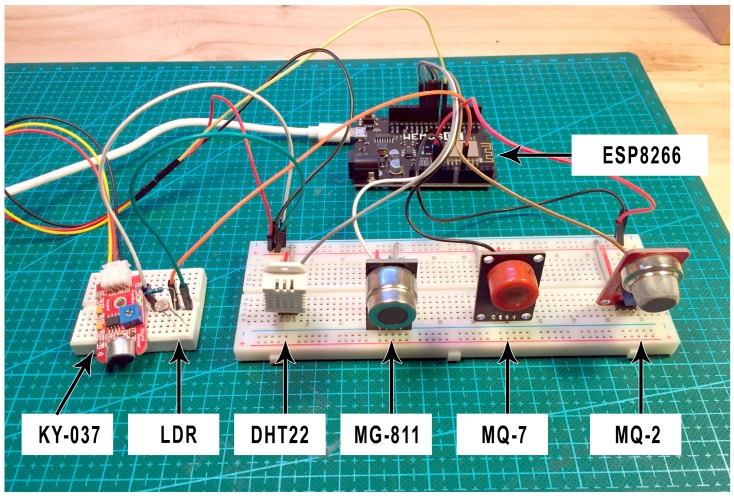
Prototype based on the Arduino board and air quality sensors.

Figure 4 shows the sensor network (MG811, MQ-7, MQ-2, and DHT22) connected to the Arduino microcontroller. Additionally, the system incorporates a sound sensor (KY-037) and an LDR sensor in order to provide context data such as the presence of people in the room or the state of light. The final set of sensors will be responsible for collecting all data in the infant’s room.

Additionally, the system will have wireless WiFi connectivity, allowing the data to be sent to the remote server. This is possible thanks to the connection of an ESP8266 module that allows for the connection of the Arduino microcontroller board with the home’s wireless network.

### 4.3. Harmful Concentration Levels

At present, there are different international agencies and societies in charge of designing and elaborating different air quality standards. These standards warn of gas concentration levels that may be harmful to health, according to the conducted medical studies. Some of the main standards are:

- NAAQS (National Ambient Air Quality Standards) [74]; this is a standard for the regulation of air quality in open spaces in the United States.
- OSHA (Occupational Safety and Health Administration) [75] is also a US standard for air regulation in industrial environments.
- MAK (Maximum Concentrations at the Workplace) [76] for the regulation of quality levels in industrial environments provided by the German government.
- Canadian [77] air quality levels for domestic and housing environments created by the Canadian government.
- WHO (World Health Organization)/Europe [78] general air quality standard (both indoor and outdoor).

One of the main societies in charge of writing and reviewing these standards is ASHRAE (American Society of Heating, Refrigerating and Air-Conditioning Engineers) [79]. This company has developed a standard called Ventilation for Acceptable Indoor Air Quality [80], which lists harmful concentration levels for common gases. It is a widely accepted standard in the scientific community and will be the basis for determining dangerous concentration levels in the proposed system. Table 3 shows the maximum exposure levels to these gases (CO, CO_2_, and tobacco smoke) during a day that will be used as reference values in the Service Provider module of our proposed BDCaM framework. The maximum humidity and temperature levels in a room where the newborn sleeps are also detailed. These data correspond to the central region of Spain and to the temperate seasons (such as spring or autumn) during which the case study described in this work was carried out.

### 4.4. Heart Rate Calculation through Open CV

Once the air quality factors in the newborn’s bedroom have been monitored, the task of obtaining the pulse through a non-invasive system is dealt with. For this purpose, we employed a technique called photoplethysmography [81]. Photoplethysmography or PPG is a technique of non-invasive detection of the cardiovascular pulse wave (also called pulse of blood volume) in the reflected light variations. This technique provides the information needed to calculate the heart rate. A small wireless webcam on the periphery of the newborn’s cradle to obtain images from which data are extracted.

One problem with the measurement of PPG is that it is susceptible to signal corruption induced by motion and motion artifacts, as detailed in [82], which are caused by the movement of whole or part of the displayed object. An optimal technique for eliminating noise in the PPG signal is the blind separation of BSS sources (Blind Source Separation) [82].

Blind source separation is a signal processing technique that consists of estimating sources in linearly mixed signals. For the treatment of the PPG signal, the BSS is implanted using the ICA technique (Independet Component Analysis) [83].

ICA is a technique of signal discovery from independent sources of a set of observations that are composed of linear mixtures of the underlying sources.

The data is represented by a random vector x (1) and the components by a random vector s (2). The objective of ICA is to transform the observed data x using a linear static transformation w as indicated in (3) in S maximally independent components, measured by some function F (4) of independence. This technique is used because it contains a large capacity to reduce artifacts by movement in PPG signals.

(1) $$x={\left(x_{1},\ldots ,x_{m}\right)}^{T}$$

(2) $$s={\left(s_{1},\ldots ,s_{n}\right)}^{T}$$

(3) s=Wx

(4) $$F\left(s_{1},\ldots ,s_{n}\right)$$

**Figure 5:**
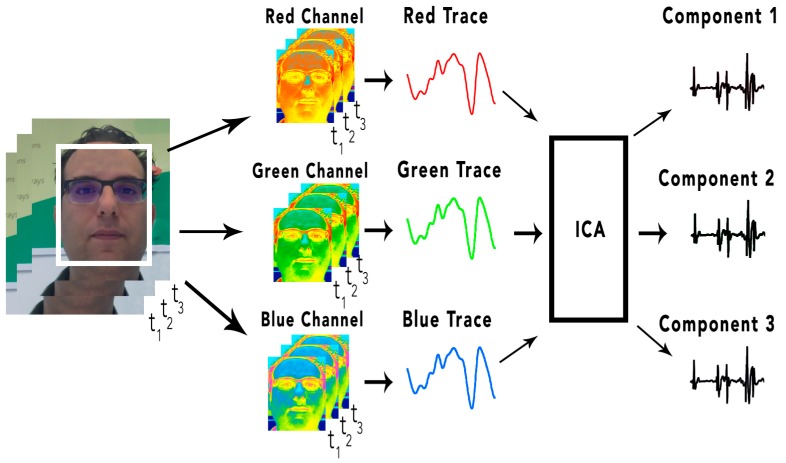
Obtaining the plethysmographic signal.

The underlying signal of interest that ICA uses is the wave of the cardiovascular pulse that spreads throughout the body. During a cardiac cycle (cardiovascular pulse), the facial blood vessels change volumetrically by modifying the length of the trajectory of the incident light; these changes in the amount of reflected light indicate the time of the cardiovascular events. Using the RGB sensors that make up a camera, a mixture of the plethysmographic signal reflected by the light can be collected. The objective of ICA is to find a separation of the PPG signal in order to collect the independent sources contained in it. An overview of how the pulse is recovered by volumetric changes in the blood vessels (plethysmographic signal) is shown in Figure 5.

First, a tracking and face detection system has been developed within the video frames to locate the region of interest. For this, OpenCV (Open Computer Vision) is used. The face detection algorithm is based on the work of the authors of [35] through a pre-trained Haardcascade capable of detecting the region of interest in an image.

Once the region of interest is located, it is separated into three RGB channels, generating a spatial average over all the pixels of the region, thus producing the three signals: Red, Green, and Blue. The signals generated are decomposed into three other signals from independent sources using ICA. Finally, the FFT (Fast Fourier Transform) is applied to the second resulting signal to obtain the power spectrum. The pulse frequency is designated as the frequency that corresponds to the highest power of the spectrum within an operating frequency band. Therefore, an operating range of [0.75, 4] Hz is used, which corresponds to [45, 240] bpm (beats per minute). In this way, a wide range of heart rate measurements is provided.

**Figure 6:**
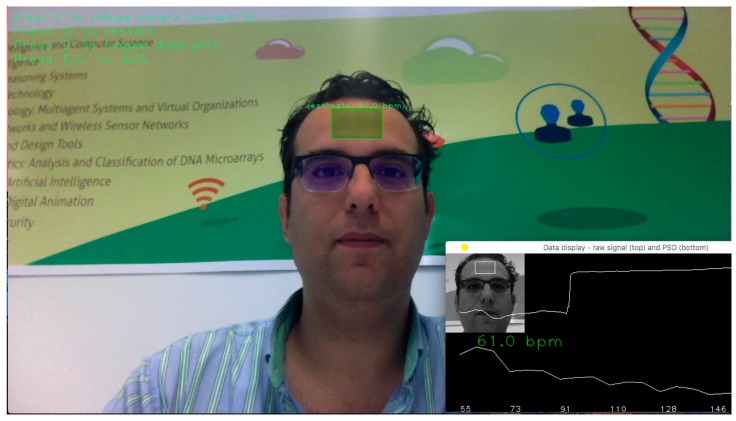
Example of the pulse measuring system in use.

Figure 6 shows the system operating through a wireless webcam. The software recognizes the region of interest that is the forehead (in green) and determines the user’s pulse through the analysis of the images.

### 4.5. Final System

**Figure 7:**
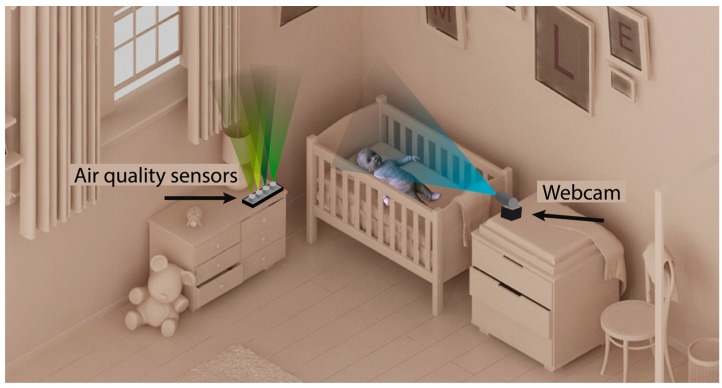
Location of the nodes in the newborn’s room.

Having described the elements of the designed system, the connection between the different nodes of the network is detailed. Figure 7 shows that the proposed system is based on the installation of the two nodes described in the previous sections. One of the nodes is the air quality sensor system shown in Figure 4, which is located next to the newborn’s cradle. It is a wireless device that can be connected to the conventional electrical network. The other node is the webcam that takes images for the calculation of the pulse; it must be located in front of the cradle. In this way, the face of the newborn is captured regardless of the position in which it is sleeping.

**Figure 8:**
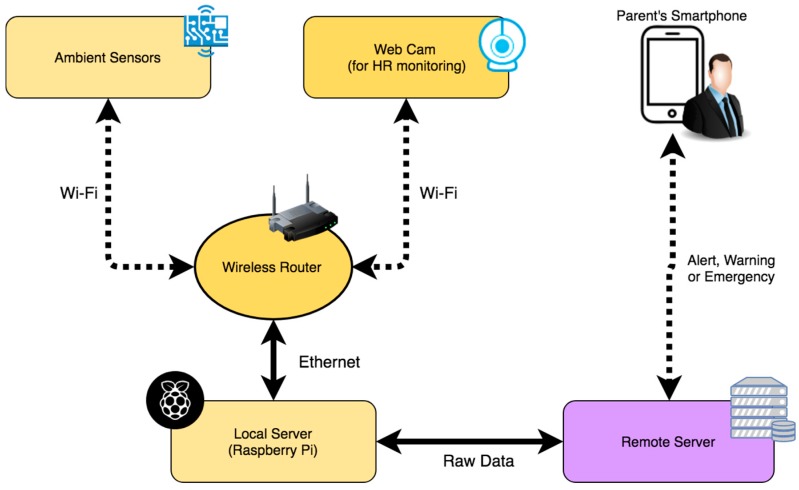
General diagram of the system.

The data captured by the different air quality sensors are sent wirelessly every 120 s through the domestic Wi-Fi network to the central node of the system. The images of the webcam are also sent to this node. Figure 8 shows the different elements of the system and their different connections in the network. As a local server that acts as the central node responsible for obtaining the information that arrives from the other two nodes, the Raspberry Pi microcomputer is used. It is a low cost device that has the necessary computing capacity both to obtain air quality data and to process the images of the webcam and get the pulse of the newborn. Once these data are analyzed, they are sent in raw to the remote server defined in the BDCaM model, which will be responsible for processing and analyzing the information. In the case of detecting an anomaly in the values obtained (such as measured values above the maximum allowed or the loss of the newborn’s pulse), the emergency process is triggered, and an alert notification is sent to the mobile device of the parent or caregiver.

### 4.6. Experimental Results

At the time of performing the experimentation of the system designed in this work in a real environment, 5 households were selected in which the system was deployed for 3 months. In the households, described in Table 4 and Table 5, neonates live between the ages of 3 and 14 months.

In order to generate specific rules for each baby within the system, the contextual data related to each one has been transformed into numerical data as shown in Table 5. Thanks to this transformation, the processing of the data and its subsequent analysis is carried out in a faster and simpler way. These data are fundamental when contextualizing the information measured by the sensorization system.

In a traditional alert system based on thresholds, there are only two possible states for an alert: active or not active. The designed system is able to identify different alert levels and trigger actions according to their degree. Table 6 shows the different classes and basic classification rules defined by the human experts for the case study. These rules are based on the current context calculated in the context-aware framework designed.

To evaluate the accuracy and performance of the system, a comparison was made between the classifications made by the proposed system and a set of general medical rules based on thresholds. In Table 7, it is possible to visualize the obtained results. Each of the measured data represents each of the measurements made by the system. As previously described, the system sends a data (set of values measured by the different sensors) every 120 s; this leaves a total of 720 data per day. Thus, during the 3-month case study 64,800 data were obtained in total from the 5 houses. After contextualizing the data, the new aggregated values are analyzed together with the original data. After preprocessing these data and eliminating erroneous data produced by system failures or connection errors, less than 64,800 data were left. In Table 7, we can observe that when a measured value is above or below the threshold, the traditional threshold-based system deduces this abnormal situation producing numerous false positives. On the other hand, the proposed system is able to learn the corrected threshold quickly for each patient and in each context.

**Figure 9:**
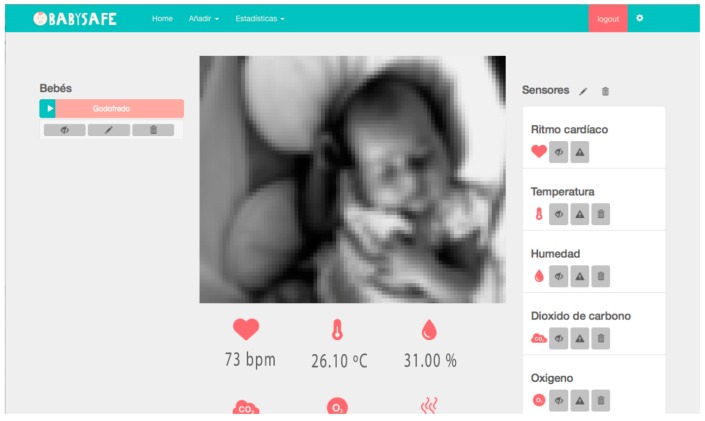
The developed web application. The image of the child has been distorted for privacy reasons.

Additionally, a web application has been developed to control and visualize all the parameters. Figure 9 shows a screenshot of the developed web interface. In the central part, the image of the baby is shown in real time. For security reasons, this functionality is only available if the user accesses the application from the same home network where the camera is located. The images taken by the camera are not sent through the network. The values measured in real time are displayed in the lower part of the image. On the right-hand side, we can find a list of the different sensors. In the lower part of the image, the icons are used to define the threshold values from which the different warnings and alerts are triggered. It is also possible to turn off or activate the sensors individually or display a history of data recorded by them.

A mobile application has been developed in order to notify the parents and caregivers of any incidents. This application consists of a simple interface which can be accessed by logging in. Once the user is logged in, the remote server will notify the user when an emergency occurs. This application, therefore, allows parents or caregivers to intervene quickly when an emergency occurs.

## 5. Conclusions

This article presents an air quality sensor system capable of monitoring the main risk factors that can trigger the Sudden Infant Death Syndrome. Thanks to the use of a context-aware framework, it is possible to generate personalized knowledge for each user, allowing for the deduction of precise rules based on the context. The aggregation of context data such as weather or air quality conditions to deployed sensorization systems allows for greater precision in the analysis. The different sensors in the system are coordinated by an Agent-based Virtual Organization, which can be deployed in each of the sensorization devices. Thanks to this coordination, the system is able to integrate a variety of sensors with different communication protocols, both wired and wireless (Bluetooth, Wi-Fi, RFID).

The proposed sensorization system is a completely non-intrusive system. Sensors are not in direct contact with the newborn, and they have no influence over its sleep. This is an important development in the monitoring of newborns, since most of the systems studied in Section 2 use intrusive sensors that impede the infant from resting properly. In this work, not only the air quality of the environment is monitored, but also the HR of the newborn is monitored with the images captured by a web camera located in front of the cradle.

The total cost of the monitoring system is below US $150, making it a low cost system that is easy to configure. Likewise, a web data visualization system has been developed that provides parents and guardians with access to all the information that is being measured in real time, the historical data, and the assessments made by the system. Moreover, an application for mobile devices has been designed. It provides information and is a fundamental tool for warning the parents when the infant is in a dangerous situation. Thanks to this, corrective actions can be carried out quickly and directly, without producing false alarms. In other SIDS monitoring systems, however, false alarms are common.

To evaluate the proposed system, a case study was carried out in the homes of 5 different infants. As demonstrated by the data obtained in the case study, the use of a framework based on context significantly reduces the number of false positives of traditional threshold-based models. These notable results aid in establishing safer environments for newborns, reducing the current incidence of SIDS. The use of context-aware frameworks provides solidity and flexibility. This is due to the integration of different technologies and protocols in combination with data contextualization techniques in which external heterogeneous data sources are used.

## Figures and Tables

**Table 1:** Comparison of context aware middleware architectures shown in [41].

Middleware	Fault Tolerance	Interoperability	Service Discovery	Storage	Context Awareness Level
CAMPH ^1^ [57]	No	No	Yes	Yes	Medium Level
ACoMS+ ^2^ [58]	data	No	Yes	Yes	Medium Level
Octopus [59]	Yes	No	No	Yes	Medium Level
CAMPUS ^3^ [60]	No	Yes	Yes	Yes	Semantic
SeCoMan ^4^ [61]	No	Yes	No	Yes	Location Aware
BDCaM [55]	Yes	Yes	Yes	Yes	Semantic
FlexRFID [62]	Yes	No	Yes	Yes	Medium Level

^1^ (CAMPH) Context Aware Middleware for Pervasive Elderly Homecare Raw data; ^2^ (ACoMS+) Autonomic Context Management System; ^3^ (CAMPUS) Context-Aware Middleware for Pervasive and Ubiquitous Service; ^4^ (SeCoMan) Semantic Web-Based Context Management.

**Table 2:** Major Risk Factors for the infants described in [73].

Risk Factor	Value
Age	≤1 year
Sex	Male
Age of the mother	≤20 year
Use of the pacifier	No
Prematurity	Yes
Position during sleep	Prona and lateral
Exposure to tobacco smoke	Yes
Surface at bedtime	Soft
Share bed with parents	Yes
Thermal stress/excess heating	Yes
Season	Cold season

**Table 3:** Maximum Concentration Levels in Indoor Environments.

Measurement	Maximum Value (Threshold)
Carbon monoxide (CO)	9 ppm
Carbon dioxide (CO_2_)	900 ppm
Smoke concentration (Tobacco)	0.40 ppm
Temperature (°C)	25 °C
Humidity (%)	60%

**Table 4:** Description of households in which the designed system was deployed.

Home	Number of Occupant	Baby Age	Shared Room	Average Temp. of the Environment
Home 1	3	8 months	No	21°
Home 2	5	10 months	Yes	22.5°
Home 3	4	14 months	No	19°
Home 4	3	3 months	No	20.5°
Home 5	6	9 months	Yes	24.5°

**Table 5:** Description of the main factors that are characteristic of the babies participating in the study.

Baby	Sex	Premature	Shared Room	Mother’s Age (≤20)	Pacifier Use	Bed Surface
Home 1	0 (Male)	0 (No)	0 (No)	0 (No)	1 (Yes)	1 (Hard)
Home 2	1 (Female)	1 (Yes)	1 (Yes)	0 (No)	1 (Yes)	1 (Hard)
Home 3	1 (Female)	0 (No)	0 (No)	1 (Yes)	0 (No)	0 (Soft)
Home 4	0 (Male)	0 (No)	0 (No)	0 (No)	1 (Yes)	1 (Hard)
Home 5	0 (Male)	1 (Yes)	1 (Yes)	1 (Yes)	1 (Yes)	0 (Soft)

**Table 6:** The different levels of problem situation based on the human expert rules.

Situation	Value	Classification Rule
Normal	0	All values are in the expected range of threshold in the current context
Warning	1	One of the values is not in the expected range, except HR in the current context
Alert	2	HR is out of the expected range or 2 or more air quality levels are out of the expected in the current context
Emergency	3	All values are out of the expected range in the current context

**Table 7:** Comparison between the proposed context-based model and threshold-based model.

		Threshold Rules		Proposed Model			
Home	Total Data	Normal	Abnormal	Normal	Warning	Alert	Emergency
1	61,298	128	61,170	43,583	13,886	3617	212
2	62,015	26	61,989	48,434	12,403	992	186
3	60,251	247	60,004	53,623	6025	501	102
4	61,891	105	61,786	56,939	4641	224	87
5	58,368	67	58,301	45,360	11,489	1318	201
